# Peripheral Blood IFN Responses to Toll-Like Receptor 1/2 Signaling Associate with Longer Survival in Men with Metastatic Prostate Cancer Treated with Sipuleucel-T

**DOI:** 10.1158/2767-9764.CRC-24-0439

**Published:** 2024-10-18

**Authors:** Michael C. Brown, Vincent M. D’Anniballe, David Boczkowski, Harini Kandadi, Nadeem Sheikh, William Kornahrens, Elisabeth I. Heath, Archana Thakur, Wei Chen, Lawrence Lum, Frank C. Cackowski, Julie Boerner, Michael D. Gunn, Andrew J. Armstrong, Smita K. Nair

**Affiliations:** 1 Department of Neurosurgery, Duke University School of Medicine, Durham, North Carolina.; 2 Department of Medicine, Duke University School of Medicine, Durham, North Carolina.; 3 Department of Surgery, Duke University School of Medicine, Durham, North Carolina.; 4 Dendreon Pharmaceuticals, LLC, Seattle, Washington.; 5 Karmanos Cancer Institute, Wayne State University, Detroit, Michigan.; 6 Division of Hematology and Oncology, University of Virginia School of Medicine, Charlottesville, Virginia.; 7 Duke Cancer Institute Center for Prostate and Urologic Cancers, Durham, North Carolina.; 8 Department of Pathology, Duke University School of Medicine, Durham, North Carolina.

## Abstract

Mounting evidence links systemic innate immunity with cancer immune surveillance. In advanced metastatic castration-resistant prostate cancer (mCRPC), Black patients have been found to have increased inflammatory markers and longer survival after sipuleucel-T (sip-T) therapy, an FDA-approved, autologous cell therapy. We hypothesized these differences may be explained by previously reported ancestral differences in pattern recognition receptor signaling, which broadly governs innate inflammation to control adaptive immune cell activation, chemotaxis, and functionality. We discovered that peripheral blood mononuclear cell IFN-β responses to Toll-like receptor 1/2 (TLR1/2), a sensor of bacterial and gut microbiome constituents, associated with significantly longer survival after sip-T therapy in two separate cohorts of men with mCRPC (discovery cohort: *n* = 106, HR = 0.12; *P* = 0.019; validation cohort: *n* = 28, HR < 0.01; *P* = 0.047). Higher IFN-β induction after TLR1/2 stimulation was associated with lower HRs than biomarkers of vaccine potency and other prognostic factors in mCRPC. TLR1/2-dependent cytokine induction was stronger in Black individuals (1.2-fold higher for IFN-β; *P* = 0.04) but was associated with survival independently of race or numbers of vaccine-induced tumor antigen–specific T cells. IFN-β responses to TLR1/2 signaling correlated with increased numbers of IFN-γ producing T cells after broad, tumor antigen–independent stimulation. Thus, peripheral innate immunity differs by race, may predict survival after sip-T, and associates with peripheral T-cell functionality in men with mCRPC.

**Significance:**

The identification of factors that determine successful cancer immunotherapy, particularly in refractory tumor types like mCRPC, is urgently needed: both to identify patients that may benefit from such therapies and to uncover routes to sensitize patients with cancer to immunotherapy. Our work links functional peripheral immune responses with race and survival after cellular immunotherapy in men with mCRPC.

## Introduction

Peripheral innate inflammation has been linked with the cancer immunotherapy outcome, including the inflammatory effects of microbiome constituents (1–4), peripheral blood composition (5, 6), and peripheral innate immune phenotypes (7, 8). We previously reported that the induction of IFN in peripheral blood after innate stimulation is associated with long-term progression-free survival after intratumor virotherapy in melanoma (9). Recent preclinical work also suggests that innate immune “training,” wherein innate immunity is poised after a prior inflammatory insult, bolsters endogenous antitumor immunity (10–13). Thus, innate immune fitness may contribute to—and/or predict the outcome of—clinical cancer immunotherapy.

Sipuleucel-T (sip-T) is an FDA-approved autologous cell vaccine against the prostatic acid phosphatase (PAP) antigen for men with metastatic castration-resistant prostate cancer (mCRPC; ref. 14). Retrospective analyses indicate that Black men with mCRPC survive longer after sip-T therapy than White men even after controlling for pretreatment clinical characteristics (15, 16). In localized disease, prostate cancer tumors from Black cohorts exhibit higher proinflammatory cytokine signatures, including that of type I IFNs (17, 18); higher plasma B-cell densities and NK cell activity (19); and elevated Th_2_ and inflammatory cytokines in plasma (20). Black men with mCRPC were found to have higher levels of Th_2_ and inflammatory cytokines (20), higher T-cell recruiting chemokines in serum (GM-CSF, CCL4, and CCL5), and relatively elevated co-stimulatory receptor expression on T cells both before and after sip-T treatment (21). Intriguingly, responses to the PAP antigen after sip-T were *lower* in cells from Black men than those in White men (20), despite anti-PAP–specific immune responses to sip-T (22–24) and sip-T vaccine product potency/quality previously being linked with improved overall survival (22). Moreover, the magnitude of the peripheral sip-T PAP antigen responses to sip-T was not associated with increased T-cell infiltration observed after therapy (25). These data indicate differences in the patterns of innate and adaptive inflammation between Black versus White patients with prostate cancer and imply survival after sip-T vaccine is influenced by factors independent of the capacity to induce antitumor (PAP)-specific adaptive immunity.

Differences in inflammation—either in the pattern or intensity—and sip-T responsiveness between Black and White men with mCRPC may be explained by distinct pattern recognition receptor (PRR) sensitivities or exposures, given the role of PRRs in dictating both innate and adaptive inflammatory responses (26). Indeed, differential type I IFN and proinflammatory responsiveness to Toll-like receptor 1/2 (TLR1/2) stimulation due to a TLR1 SNP I602S linked to Neanderthal admixture explained broad differences in inflammatory sensitivities between individuals from European versus African descent (27, 28). TLR1/2 recognizes bacterial triacyl lipoproteins and is a key sensor and regulator of the gut microbiome (29–31). Ligands of TLR1/2 have been identified in tumors (32, 33), and bacteria have been reported to colonize diverse tumor types (34), including prostate cancer (35), which may trigger TLR1/2 signaling. Thus, we hypothesized that ancestral differences in response to TLR1/2 ligands, due to the TLR1 I602S SNP or other factors, may influence immunotherapy outcome and associate with Sip-T efficacy. Using *in vitro* stimulation assays and genotyping of peripheral blood, we tested whether peripheral blood mononuclear cells (PBMC) inflammatory responses to *in vitro* innate immune challenge and/or TLR1/2 SNP status associate with post–sip-T survival in men with mCRPC.

## Materials and Methods

### Study design

Peripheral blood samples collected from men with mCRPC were obtained after written informed consent under approved Institutional Review Board protocols as a part of the PRIME study (NCT01727154; ref. 20) or through Wayne State University (21). These studies were conducted in compliance with the Declaration of Helsinki and US common rule. De-identified PBMCs were subject to *in vitro* stimulation assays and linked with survival outcomes and other clinical features under a protocol approved by the Duke Institutional Review Board. A subset of patients with mCRPC enrolled in PRIME subcohort (20) of the PROCEED sip-T study (16) with available banked PBMCs, self-reported race, and survival information, were included in PBMC function and flow cytometry analyses (*n* = 106; 91 White and 15 Black). Of those, 103 had sufficient DNA isolated for TLR1 SNP analysis by quantitative PCR (88 White and 15 Black). PBMCs were collected at baseline (*n* = 14), 6 weeks after therapy (*n* = 50), or 6 to 39 weeks after therapy (*n* = 42). PBMC function (cytokine secretion) using an *in vitro* stimulation assay, flow cytometry, and TLR1 SNP analyses was performed blinded to survival outcomes. A cohort from Karmanos Cancer Institute (KCI; ref. 21) comprised of pre–sip-T PBMCs from patients with mCRPC (*n* = 28, *n* = 14 White and *n* = 14 Black) was similarly tested to validate associations between PBMC *in vitro* stimulation assay outputs with survival. Separate measures from the PRIME subcohort patients were previously performed by Dendreon Pharmaceuticals, LLC and provided for this study, including ELISpot [CEFT, Fluzone, PA2024 (the vaccine antigen), PAP, and PHA response spot counts], ELISA [PA2024 IgG, PAP IgG, Tetanus IgG, PA2024 IgG–IgM, and PAP IgG–IgM], vaccine manufacture parameters [CD54 upregulation (upreg), and total nucleated cells (TNC)]. See Supplementary Fig. S1A and S1B for the CONSORT diagram explaining the discovery and validation cohorts, respectively, included in the present retrospective analysis. Supplementary Table S1 provides relevant clinical information.

### *In vitro* stimulation assay

Prior to thawing of PBMCs, 24-well plates (Griener Bio-One) were plated with 250 μL RPMI-10 media [10 mL RPMI 1640 (Gibco) supplemented with 10% FBS (Sigma-Aldrich)] containing nothing (mock), 5 μg high molecular weight (HMW) Poly I:C-LyoVec (InvivoGen), 0.5 μg lipopolysaccharide (LPS, InvivoGen), 5 ng PAM_3_CSK_4_ (InvivoGen), or 1 μg/mL anti-CD3 (RRID: AB_11150592), and anti-CD28 (RRID: AB_11148949) Ultra-LEAF antibodies (BioLegend) with 4 μg/mL of goat anti-mouse secondary antibody (Jackson ImmunoResearch; RRID: AB_2338447). PBMCs were thawed and 300 μL of the 1 mL vial were retained and processed for DNA isolation using the All-Prep DNA/RNA micro kit (Qiagen) per manufacturer’s instructions. The remaining contents were diluted in 9 mL AIM-V media (Gibco) without additives, centrifuged to cell pellets, and reconstituted in 1 mL of AIM-V containing DNAse I (10 μg/mL, Roche), followed by incubation at 37°C for 10 minutes. Aliquots of cell suspensions were counted using a Countess II automated cell counter (Thermo Fisher Scientific). Cell counts were normalized to 1.5 × 10^6^ cells/mL in 2.5 mL of RPMI-10, and 250 μL of cells was added to 24-well plates in each preplated treatment condition (six wells per sample) and incubated for 24 hours at 37°C. Cell supernatants were retained and frozen at −80°C for downstream cytokine analysis.

### Flow cytometry and supernatant cytokine measurements

Remaining PBMCs from the *in vitro* stimulation assay were washed in PBS and incubated in PBS (Gibco) containing 2% FBS with antihuman TruStain Fc-block (1:50, BioLegend; RRID: AB_2818986). After 15 minutes, cells were stained with the following antigen-specific antibodies: CD8-BUV395 (1:250; RRID: AB_2722501) from BD Biosciences; or CD4-BV510 (RRID: AB_2561866), CD3-FITC (RRID: AB_2564148), CD19-BV711 (RRID: AB_2562065), CD14-BV421 (RRID: AB_10959324), CD11b-APC (RRID: AB_312795), and CD11c-APC-cy7 (RRID: AB_10662746) at a 1:250 dilution from BioLegend for 1 hour at room temperature. Cells were washed in 1 mL PBS + 2% FBS and resuspended in PBS + 2% FBS containing 7-AAD (1:100, BioLegend). Cells were analyzed on a Fortessa X20 (BD Biosciences) at the Duke Cancer Institute Flow Cytometry Core Facility, and analyses were performed using Flow Jo v.10 (BD Biosciences; RRID: SCR_008520). Gating was guided using established negative control populations for each antigen. Collected supernatant was analyzed using the human Anti-Virus LEGENDPlex kit (BioLegend) following manufacturer instructions. Data were collected on a Fortessa X20 and analyzed using the manufacturer’s analysis software to determine median fluorescence intensity for each bead population. Fold mock control values were derived from median fluorescence intensity values to relative to patient-specific mock controls and used for downstream comparisons.

### Genotyping of TLR1 polymorphisms

Genomic DNA was analyzed for TLR1 (I602S) polymorphisms using PCR-RFLP as previously described (bioRxiv 2018.01.18.239129). In short, a fragment containing exon 4 of *tlr1* was amplified from DNA isolated from PBMCs using the following primers: forward: CTTGATCTTCACAGCAATAAAATAAAGAGCATTCC and reverse: GGCCATGATACACTAGAACACACATCACT. The PCR conditions were initial denaturation at 95°C for 5 minutes, followed by 32 cycles: denaturation at 95°C for 30 seconds, annealing at 60°C for 30 seconds, and elongation at 72°C for 32 seconds. The final elongation was at 72°C for 5 minutes. The amplicon was digested by PstI to discriminate between the *tlr1* 602 alleles. These fragments were analyzed by electrophoresis on 2.5% agarose gels stained with GelGreen Nucleic Acid Stain (Sigma-Aldrich).

### Statistical analysis

The present analysis is considered a *post hoc* exploratory analysis of clinical cohorts of men with mCRPC undergoing standard-of-care sip-T therapy. All descriptive statistical analyses were performed in GraphPad Prism v10 (RRID: SCR_002798). The primary objective was to evaluate the association between PBMC PRR responses and overall survival, defined as the time from sip-T therapy initiation to death due to any cause. A one sample *t* test (two-tailed, vs. 0) was used to determine cohort-level cytokine induction after normalization of values to log (fold mock control) for each stimulant and cytokine. Comparisons of survival were performed using a Mantel–Cox log-rank test. HRs and 95% confidence intervals (CI) from Kaplan–Meier curves were calculated using the Mantel–Haenszel test. Cox proportional hazards models were performed using graph pad Prism v10. Comparisons between two groups (e.g., by survival status, SNP status, or race) were performed using two-tailed unpaired *t* tests. Adjustment for multiple comparisons were made using the FDR (Benjamini–Hochberg method) as indicated in figure legends.

### Data availability

Data generated in this study are available upon request to the corresponding author.

## Results

### PBMCs from men with mCRPC mount varied responses to stimulation

**Figure 1:**
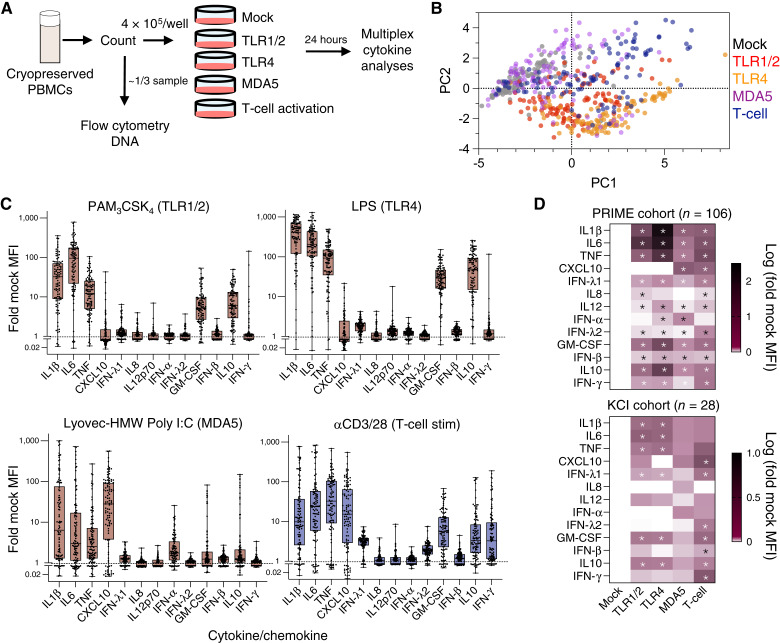
Study design and *in vitro* responsiveness of PBMCs from patients with mCRPC to PRR agonist prior to sip-T treatment. **A,** Banked pretreatment PBMCs from the PRIME sip-T discovery cohort (*n* = 106) or KCI validation cohort (*n* = 28) were subjected to DNA isolation, flow cytometry, and/or testing in an *in vitro* stimulation assay. Two hundred thousand PBMCs per well were plated and treated with no stimulation (mock) or stimulation (stim) with TLR1/2 (PAM_3_CSK_4_), TLR4 (LPS), MDA5 (Poly I:C-LyoVec), or T-cell [anti (α)-CD3/28 antibodies] after 24 hours of culture. **B–D,** Supernatant cytokine release was measured after *in vitro* stimulation. **B,** Principal component analyses colored by treatment using raw MFI values for each analyte, each data point represents a single patient. **C,** Box and whisker plots (Boxes: median ± 25%–75% quartiles, whiskers = range) of fold mock MFI values for each cytokine in the PRIME study (*n* = 106), each data point represents a single patient. **D,** Heatmaps of log (fold mock control) values are shown for the PRIME and KCI (validation) cohorts; asterisks denote FDR adjusted *Q* < 0.05 using one sample *t* test vs. 0 (no induction). MFI, mean fluorescence intensity.

Pretreatment PBMCs from men in the PRIME cohort (20) with mCRPC prior to sip-T (*n* = 106; *n* = 15 Black, *n* = 91 White) were used to test if peripheral immune function may associate with sip-T therapy survival outcomes (Fig. 1A). All data collection and analyses were performed blinded to survival outcomes in this study. We isolated DNA for downstream TLR1 SNP analysis and performed basic flow cytometry characterization on a portion of PBMCs (1/3 of the sample). An *in vitro* PBMC stimulation assay was also applied to the remaining PBMC fraction after cell count normalization. The PBMC stimulation assay tested diverse stimuli targeting the following receptors: TLR1/2 (PAM_3_CSK_4_), TLR4 (LPS), MDA5 (LyoVec complexed HMW Poly I:C for intracellular delivery), and anti-CD3/CD28 (T-cell stimulation). We selected TLR4 and MDA5 agonist for comparison to that of TLR1/2 as these PRRs use distinct signaling transducers that represent shared signaling adaptors used by other TLRs/PRRs to induce inflammatory responses (TLR1/2: MyD88, TLR4: MyD88 and TRIF, MDA5: MAVs); CD3/28 stimulation was used to specifically account for differences in T-cell function due to their role in mediating antitumor functions after sip-T vaccination. Twenty-four hours later, supernatant cytokine release was measured and normalized by dividing by patient-specific mock treatment control values. By principal component analysis, TLR1/2 and TLR4 responses were more similar and were generally distinct from MDA5 and T-cell stimulation (Fig. 1B), as expected due to their common engagement of MyD88. Indeed, TLR1/2 and 4 agonists primarily induced IL1β, IL6, and TNF, whereas LyoVec-conjugated HMW Poly I:C induced relatively higher levels of CXCL10 and IFN-α, consistent with MDA5 signaling (Fig. 1C; refs. 9, 36). T-cell stimulation via CD3 and CD28 ligation induced higher levels of IFN-γ relative to the other treatments as expected (Fig. 1C). Despite differences in the induction of specific cytokines between different stimuli, most of the tested cytokines were significantly induced (fold mock treatment) at the cohort level for the PRIME cohort (Fig. 1D; PRIME: 10/13 tested were induced by all treatments). Patterns of cytokine induction were also consistent in a separate, smaller validation cohort (KCI, *n* = 28; Fig. 1D).

### Patients with mCRPC with stronger IFN-β responses to TLR1/2 stimulation survive longer after sip-T therapy

**Figure 2:**
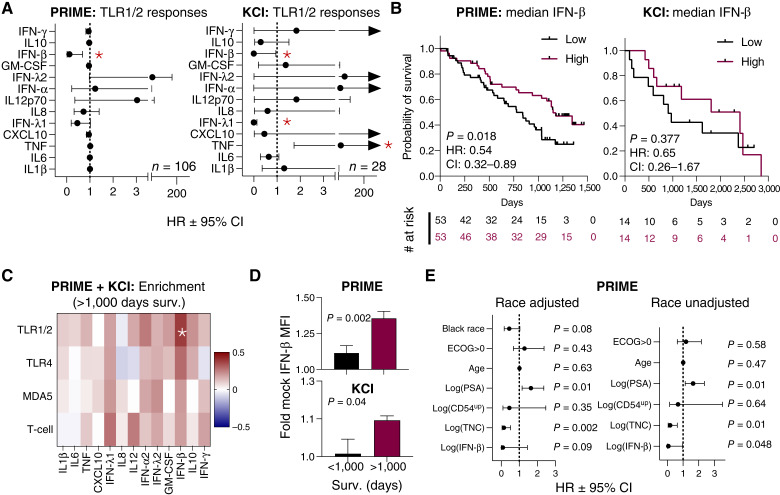
Stronger IFN-β responses to TLR1/2 stimulation associates with longer survival after sip-T therapy. **A,** HRs ± 95% CIs from a Cox proportional hazards model comparing induction of each cytokine (fold mock control) after TLR1/2 stimulation with PAM_3_CSK_4_ for PRIME cohort (left) or KCI cohort (right) vs. survival; *, *P* < 0.05; arrowheads indicate outlier upper bound CI (KCI cohort only). Supplementary Figure S2A depicts Cox proportional HRs for all treatments and cytokines. **B,** Survival of patients tested for TLR1/2 responsiveness stratified by median IFN-β induction for PRIME (left) and KCI (right) cohorts; *P* values are from Mantel–Cox test, HR and 95% CIs are from Mantel–Haenszel test. **C,** Mean *z*-scores normalized for each cytokine and treatment were computed for the combined cohort of patients (PRIME and KCI, *n* = 108), excluding patients that were censored prior to 1,000 days due to incomplete follow-up. *Z*-scores for patients surviving >1,000 days (*n* = 47) are shown and reflect enrichment/depletion relative to the broader cohort; asterisk indicates FDR adjusted (within each stimulant group) *t* test *Q* < 0.05. **D,** Fold mock treated MFI values for IFN-β after treatment with TLR1/2 stimulation comparing patients surviving < or > 1,000 days from PRIME (top) or KCI (bottom) subcohorts from **C**; *P* values are from unpaired *t* test and mean + SEM is shown. **E,** Cox proportional hazards model derived HRs ±95% CI and *P* values for indicated prognostic features and IFN-β. PSA values were not available for two patients, which were excluded from these analyses (*n* = 104). See Supplementary Figs. S2 and S3 for extended data. MFI, mean fluorescence intensity.

We next asked if PBMC responses to stimuli associated with longer overall survival in men with mCRPC after sip-T treatment in each patient cohort. Amongst all stimuli and cytokines, IFN-β induction after TLR1/2 stimulation was significantly associated with a lower HR after sip-T therapy in both the discovery cohort (PRIME, *n* = 106, HR = 0.12; *P* = 0.019) and the validation cohort (KCI, *n* = 28, HR < 0.1; *P* = 0.047; Fig. 2A; Supplementary Fig. S2). We also observed longer survival of patients with >median IFN-β induction relative to patients with below median induction in both cohorts (Fig. 2B). Conversely, we determined relative cytokine induction of long-term (>1,000 days, *n* = 47) versus short-term (<1,000 days, *n* = 61) survivors using *z*-score normalized fold mock control cytokine values merged from both cohorts; patients with <1,000 days follow-up (*n* = 26) were excluded from this analysis. We chose 1,000 days as a threshold based upon median survival of the PROCEED cohort (921 days; ref. 16), and the high number of patients censored after 1,000 days in our study cohorts. IFN-β responses to TLR1/2 stimulation were significantly enriched in long-term survivors of both cohorts (Fig. 2C and D). Notably, PBMCs from the KCI cohort were exclusively collected at baseline (pretreatment), and a survival difference by IFN-β induction was apparent regardless of pre- versus post-treatment in the PRIME cohort (Supplementary Fig. S3A). Thus, baseline IFN-β responses to TLR1/2 (prior to sip-T therapy) in PBMCs may predict improved long term overall survival after sip-T therapy.

Cumulative levels of TNC and CD54 upreg—the latter being associated with monocyte activation (22)—in the autologous monocyte vaccine product; age (37, 38); serum PSA (16, 39); and Eastern Cooperative Group (ECOG) performance scores (16) have been shown to associate with survival after sip-T and/or are known prognostic factors in mCRPC. Within the PRIME cohort, IFN-β induction after TLR1/2 stimulation was associated with a favorable HR that either approached statistical significance with adjustment for race (HR < 0.1, 95% CI, 0.01–1.46; *P* = 0.084) or remained significant without adjustment for race (HR < 0.1, 95% CI, 0.003–0.97; *P* = 0.048) when adjusted for TNC, CD54 upreg (CD54^up^), age, PSA, and ECOG performance scores (Fig. 2E). Age and PSA levels were not correlated with IFN-β induction (Supplementary Fig. S3B), and stratification by other features tested in Fig. 2E did not reveal statistically significant differences in survival after stratification (Supplementary Fig. S3C). As previously reported, higher serum PSA was associated with a higher HR, and higher TNC was associated with a lower HR in each context (16, 22). Although blood lactate dehydrogenase, hemoglobin, and alkaline phosphatase levels are also associated with outcomes in mCRPC (16), these values were only available for a subset of patients in the cohort and thus were not included in the analysis. These data imply that the association of IFN-β responses to TLR1/2 stimulation with survival is unrelated to vaccine potency (CD54 upreg and TNC), extent of disease burden at the time of treatment (PSA and ECOG), and age and may be weakly linked to race.

### IFN-β induction after TLR1/2 stimulation associates with IFN-γ responses in T cells

**Figure 3:**
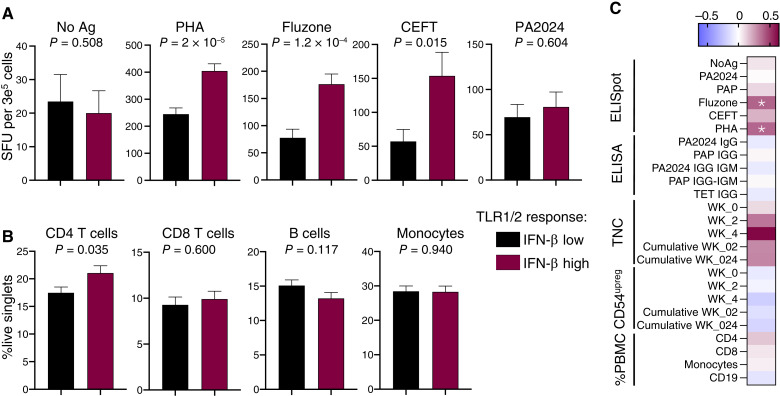
IFN-β induction after TLR1/2 stimulation associates with PBMC ELISpot responses to Fluzone, PHA, and CEFT (cytomegalovirus, EBV, Fluzone, and Tetanus peptide pool). **A,** ELISpot SFU after each indicated stimulant (*n* = 77 CEFT, *n* = 79 Fluzone, *n* = 82 for others). **B,** Cell type–specific PBMC densities as a percentage of live single cells (bottom) separated by median IFN-β response after TLR1/2 stimulation (*n* = 106, PRIME study). **A** and **B,***P* values are from unpaired *t* test; mean + SEM is shown. **C,** Correlation (Spearman *r*) of each indicated PBMC (ELISpot), serum (ELISA), or sip-T product metric (CD54 upreg and TNC) output with fold mock IFN-β values after TLR1/2 stimulation; (*) indicates FDR adjusted *Q* < 0.05. See Supplementary Figs. S4 and S5 for extended data and representative gating. SFU, spot forming units.

We next explored the associations between IFN-β induction after TLR1/2 stimulation and other immunologic features in men with mCRPC undergoing sip-T therapy. Intriguingly, although higher than median TLR1/2-induced IFN-β was not associated with higher numbers of the sip-T vaccine antigen PA2024/PAP-specific T cells after treatment, it was associated with increased spot counts after tumor antigen independent stimulation, including after PHA, Fluzone, and CEFT stimulation (Fig. 3A; Supplementary Fig. S4A). Notably, IFN-β responses were also associated with higher proportions of CD4^+^ T cells (Fig. 3B; Supplementary Figs. S4B, S4C, and S5). However, the relationship between IFN-β responses and IFN-γ spot counts after PHA, Fluzone, and CEFT remained after normalizing the ELISpot spot counts by CD4 T-cell percentages (of total live PBMCs, Supplementary Fig. S4D). Moreover, Fluzone- (Spearman *r* = 0.39, *Q* value 0.004) and PHA (Spearman *r* = 0.40, *Q* value 0.004)-induced IFN-γ T-cell spot counts significantly correlated with IFN-β induction, whereas CD4 T-cell levels did not (Fig. 3C). Lastly, IFN-β induction remained significantly associated with a lower HR after adjusting for PBMC cell type proportions (Supplementary Fig. S4E). Overall levels of IFN-γ induction—as opposed to the number of cells secreting IFN-γ—after T-cell stimulation (using CD3/28 ligation, Fig. 1) also correlated with TLR1/2-induced IFN-β (Spearman *r* = 0.22, FDR adjusted *P* = 0.04; Supplementary Fig. S4F) but was not as robust as the correlation between TLR1/2-induced IFN-β versus IFN-β induced by other treatments (Supplementary Fig. S4F). Collectively, our data indicate that rather than predicting numbers of antitumor (PAP/PA2024) vaccine–specific T cells, TLR1/2-induced IFN-β responses associate with the functional capacity and/or proportion of T cells that secrete IFN-γ expression after stimulation.

### Cytokine responses to TLR1/2 stimulation are stronger in Black patients with mCRPC and patients with the TLR1 602 I/I or I/S polymorphisms

**Figure 4:**
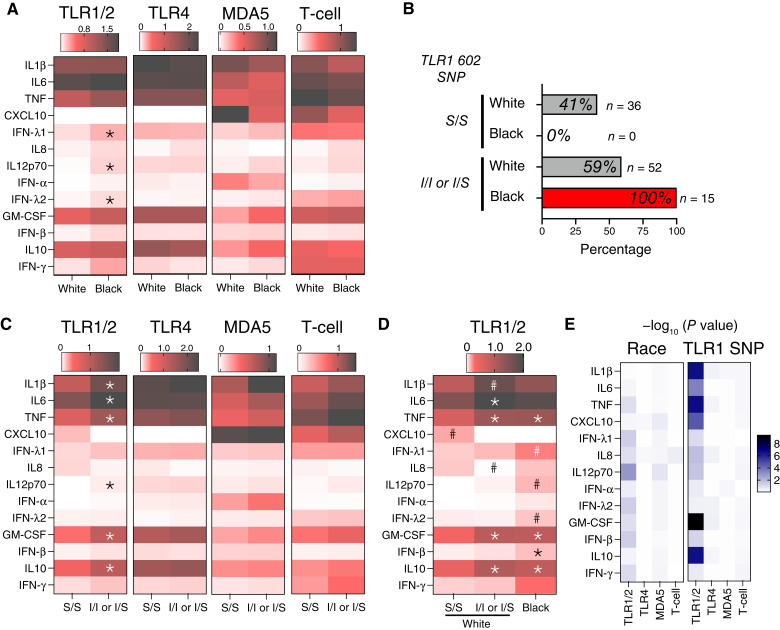
Black race and TLR1-602I SNP associate with stronger responses to TLR1/2 stimulation. **A,** Supernatant cytokine release separated by Black (*n* = 15) vs. White (*n* = 91) for each stimulation normalized to fold mock treatment control in the PRIME study; (*) Mann–Whitney test *P* < 0.05. **B,** TLR1-602 genotypes for each patient with sufficient DNA (*n* = 88 White; *n* = 15 Black) were tested by PCR-RFLP; percentages of I/I or I/S vs. S/S by race are shown. **C,** Mean fold mock control cytokine induction by genotype is shown for each stimulus; asterisks indicate significant FDR-corrected unpaired *t* tests *Q* < 0.05 (two-tailed). **D,** Mean fold mock control TLR1/2 induced cytokine induction by genotype for White vs. Black (all I/I or I/S); FDR-adjusted unpaired *t* test *Q* < 0.05 vs. White S/S (*) or vs. all other groups (#). **E,** Heatmaps depict −log (*P* values) from comparisons in **B** (cytokine induction by race) or **C** (cytokine induction by TLR1 SNP status) for each cytokine and treatment. See Supplementary Fig. S6 for extended data.

Prior reports indicate that African ancestry is associated with greater sensitivity to TLR1/2 ligand stimulation (27, 40). Indeed, proinflammatory cytokine release after TLR1/2 stimulation was overall stronger in Black individuals with mCRPC in the PRIME dataset as compared with that of White individuals, with significantly higher IFN-λ1/2 and IL12 responses (Fig. 4A; Supplementary Fig. S6A and S6B). Notably, IFN-β induction was significantly higher in Black individuals prior to adjustment for multiple comparisons (*P* = 0.04; *Q* = 0.1). Statistically significant differences in cytokine secretion by race were only observed after TLR1/2 stimulation. Differences in ELISpot and ELISA based responses against vaccine antigen/other antigens were also not observed by race, except for lower PA2024 IgG + IgM and a trend toward lower PA2024 T cells in Black patients (Supplementary Fig. S6C–S6E). Black individuals also had higher CD4 and CD8 T cells, but lower B cells and monocytes as a proportion of total PBMCs (Supplementary Fig. S6F).

Enhanced TLR1/2 signaling in PBMCs from individuals of African ancestry has been shown to be largely determined by the SNP ile(I)602-to-ser(S) in exon 4 of *tlr1*, with the I/I and I/S genotypes associated with stronger responses to TLR1/2 stimulation (27). PCR-RFLP of *tlr1* exon 4 determined that TLR1 602 I/S or I/I genotype was present in 100% and 59% of Black and White individuals in the mCRPC PRIME cohort (Fig. 4B), respectively, consistent with the previously observed distributions (27). Stratifying cytokine induction by TLR1 genotype confirmed the association of TLR1602 I/S or I/I with enhanced responsiveness to PAM_3_CSK_4_-based TLR1/2 stimulation (27), with no significant differences observed for other tested stimuli (Fig. 4C). Stratification of White versus Black patients by genotype confirmed that in White individuals with I/S or I/I genotypes, stronger responses to TLR1/2 versus that of S/S occurred, with exception of CXCL10 and IL8 (Fig. 4D). However, relative to White individuals regardless of genotype, Black individuals also had higher IFN-λ1/2, IL12, and IFN-β induction (Fig. 4D and E), possibly indicating other factors drive stronger or divergent responses to TLR1/2 than the TLR1 602 SNP.

### The association of pretreatment IFN-β responses with survival after sip-T therapy is independent of race and TLR1 SNP status

**Figure 5:**
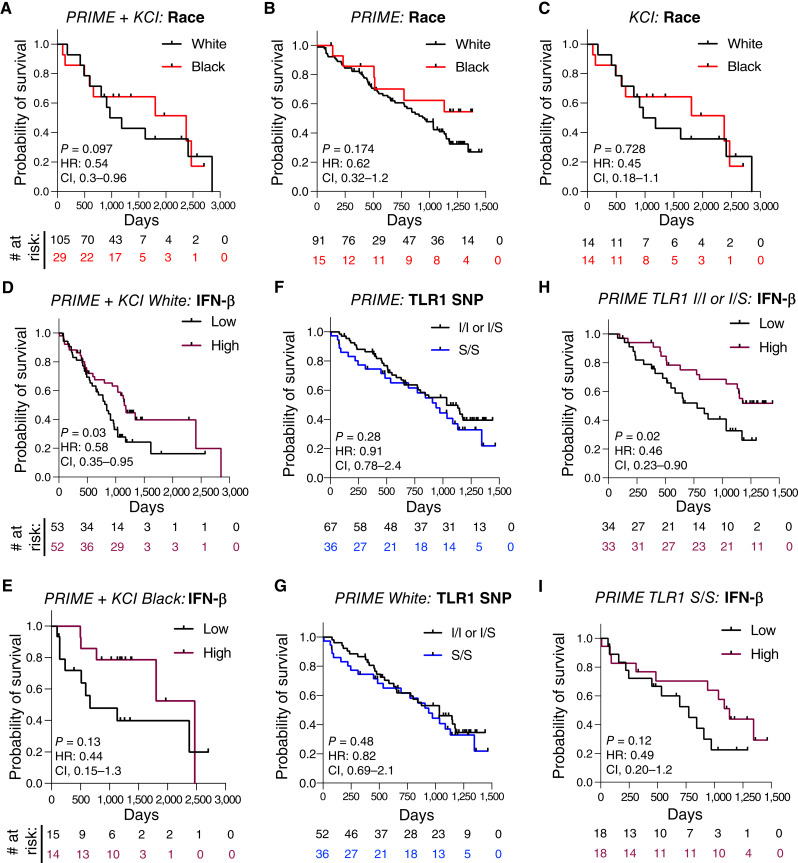
TLR1/2 responses associate with post–sip-T survival independent of race and TLR1/2 SNP status. **A–C,** Survival stratified by race in the merged PRIME and KCI cohorts (**A**), PRIME cohort alone (**B**), or KCI cohort alone (**C**). **D** and **E,** Survival of White (**D**) or Black (**E**) patients from the merged (PRIME and KCI) cohorts stratified by respective cohort median (PRIME or KCI) IFN-β induction after TLR1/2 stimulation. **F** and **G,** Survival of the PRIME cohort (TLR1 SNP genotyping was not performed in the KCI cohort) stratified by TLR1 602 I/I or I/S vs. TLR1 602 S/S for all races (**F**) or for White patients (**G**). **H** and **I,** Survival of patients with TLR1 602 I/I or I/S SNP (**H**) or TLR 602 S/S (**I**) stratified by median IFN-β induction after TLR1/2 stimulation. HRs and 95% CI are from the Mantel–Haenszel test; *P* values are from the Mantel–Cox log-rank test. See Supplementary Fig. S7 for extended data.

In line with effects observed in larger cohorts of sip-T–treated patients, Black patients generally survived longer than White counterparts in both study cohorts (Fig. 5A–C), although not statistically significant likely due to sample size limitations. The induction of IFN-β after TLR1/2 stimulation was higher in Black patients (Fig. 4), implying that race/ancestry may explain this association with longer survival. However, higher than median IFN-β induction was consistently associated with longer survival both in White patients (Fig. 5D; Supplementary Fig. S7A) and Black patients (Fig. 5E). TLR1 genotype was not associated with longer survival in the total cohort (White + Black, Fig. 5F) or in the White subcohort (Fig. 5G), and IFN-β responses were associated with longer survival regardless of TLR1 genotype (Fig. 5H and I; Supplementary Fig. S7B). Thus, we conclude that other factors beyond TLR1 genotype (e.g., other genetic determinants, microbiome, innate immune training, or environmental differences) explain the variation in IFN-β responses after PBMC stimulation and its relationship with survival after sip-T therapy.

## Discussion

This work identified an unexpected link between the sensitivity of pretreatment peripheral TLR1/2 signaling and IFN-β responsiveness with overall survival after sip-T immunotherapy in men with mCRPC. The impetus for this study was the hypothesis that established differences in PRR signaling, which shape both innate and adaptive inflammation, in individuals with African ancestry (27) may explain the longer observed survival of Black patients with prostate cancer after sip-T therapy in several prospective clinical trials (14–16). Indeed, relatively higher IFN-β secretion by pretreatment PBMCs after TLR1/2 stimulation was consistently associated with a lower HR than other known predictive/prognostic factors including PSA levels, features of vaccine product potency [TNC, cumulative CD54 upreg, and tumor antigen (22)], and age (15). Although we confirmed Black individuals mounted stronger inflammatory responses to TLR1/2, the association between survival and IFN-β secretion after TLR1/2 stimulation was observed irrespective of race and TLR1/2 genotype, indicating a polygenic or gene–environment interaction that may explain race/ancestry associations with TLR1/2 signaling, IFN-β response, and improved survival with sip-T in men with mCRPC.

It remains to be determined why peripheral TLR1/2 responsiveness is associated with survival after sip-T and whether it is truly predictive of survival after sip-T therapy or is generally a prognostic feature in men with mCRPC irrespective of therapy given. Moreover, other confounding variables (e.g., corticosteroid use) that we were unable to control for in the current study may influence this association, although differences in IFN-β induction after stimulation with other innate stimulants were not associated survival, implying that general immunosuppression is unlikely to explain our observations. Future race-inclusive and PBMC biomarker defined studies of other non–sip-T–treated cohorts of men with mCRPC should address these questions. We speculate that sensitivity to TLR1/2 ligands may lead to enhanced intratumor and/or systemic inflammation triggered by endogenous TLR1/2 ligands [e.g., the gut microbiome (29), tumor microbiome (34, 35), and/or endogenous “self” ligands within the tumor (32, 33)] that could conceivably induce chemokine secretion within the tumor to enable trafficking of antitumor T cells to the tumor site, enhance antitumor T/B-cell functionality, and/or activate tumor local antigen presenting cells. Indeed, work from the broader PRIME (20) and KCI (21) cohorts represented in this study discovered higher levels of inflammatory cytokines/chemokines in Black men with mCRPC treated with sip-T, consistent with our findings. Antitumor T-cell responses against the vaccine antigen PA2024 were higher in patients living longer after sip-T (20, 24). However, the induction of IFN-β after TLR1/2 stimulation was not associated with quantities of vaccine-mediated antigen-specific T cells or antibodies (PA2024 or PAP) but rather was associated with quantities of T cells responding to tumor antigen–independent stimuli (PHA, CEFT, and Fluzone). This distinction may reflect greater systemic T-cell functionality, and if so, would imply that IFN responses to TLR1/2 are associated with adaptive immune fitness. Lastly, Black men with prostate cancer, which we found are more sensitive to TLR1/2 stimulation, have higher inflammatory signatures—including that of type I IFNs— in their tumors (17). The extent to which TLR1/2 signaling may contribute to these differences remains to be determined.

Although we validated the relationship between TLR1/2-induced IFN-β and survival after sip-T in an independent cohort of men with mCRPC, these results require further validation in a larger cohort of patients, ideally in a prospective, adequately powered and race-stratified manner to enable real time immune phenotyping over time. Moreover, the cohort tested was not sufficiently powered to explore the observed longer survival of Black individuals relative to White individuals after sip-T, and its potential association with TLR1/2 responsiveness and other features, due to the limited number of Black patients in each cohort (PRIME: *n* = 15; KCI: *n* = 14). Although we performed basic flow cytometry analysis on PBMCs prior to treatment and conducted two independent measures of viability (trypan blue exclusion and 7-AAD staining), it is possible that a subset of cell types may explain differential IFN-β induction after TLR1/2 stimulation and that differences in PBMC quality may influence these results (e.g., plasmacytoid dendritic cells). However, responses after stimulation of other PRRs (LPS, MDA5, and anti-CD3/28) did not associate with survival, suggesting that differences in PBMC quality or composition do not explain our observations. Lastly, although our study focused primarily on IFN-β induction after TLR1/2 stimulation due to its consistent association with survival after sip-T therapy, it must be noted that other cytokines had similar, but statistically weaker, associations with survival. Among these include IFN-λ2, which was nonsignificantly associated with shorter survival, and IFNλ1, which was nonsignificantly associated with longer survival. Further investigation into the patterns of inflammation and their impact on immunotherapy outcomes are warranted.

Together, our observations add to mounting evidence indicating the potential of ancestry-associated peripheral inflammation in predicting survival after cancer immunotherapy and may imply that the functionality of the peripheral immune system influences cancer immunotherapy efficacy. The observation that peripheral immune status associates with cancer immunotherapy outcomes may also indicate that augmenting systemic innate immune could be used to sensitize patients to cancer immunotherapy.

## Supplementary Material

Table S1 Clinical cohort characteristics

Figure S1 Consort diagram

Figure S2 Related to Figure 2

Figure S3 Related to Figure 2

Figure S4 Related to Figure 3

Figure S5 Related to Figure 3

Figure S6 Related to Figure 4

Figure S7 Related to Figure 5
