# Supplementary material for: Peripheral Blood IFN Responses to Toll-Like Receptor 1/2 Signaling Associate with Longer Survival in Men with Metastatic Prostate Cancer Treated with Sipuleucel-T
Source: Cancer Res Commun. 2024 Oct 18;4(10):2724–33. doi: 10.1158/2767-9764.CRC-24-0439 (PMC11487532; doi:10.1158/2767-9764.CRC-24-0439)
Supplement: Figure S1 — Consort diagram [file crc-24-0439_figure_s1_suppsf1.pptx]

## Slide 1
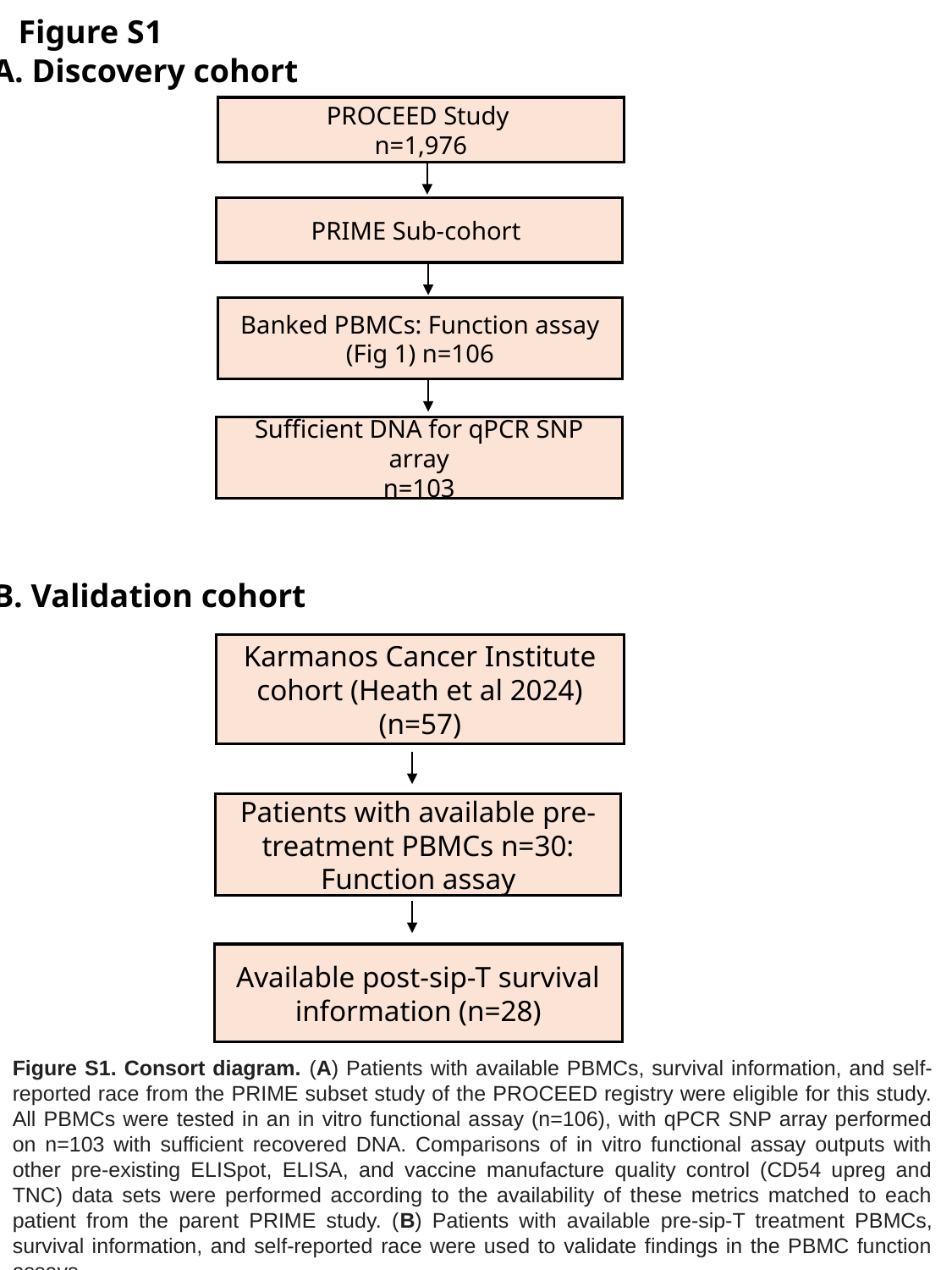

Figure S1
A. Discovery cohort
PROCEED Study
n=1,976
PRIME Sub-cohort
Banked PBMCs: Function assay (Fig 1) n=106
Sufficient DNA for qPCR SNP array
n=103
B. Validation cohort
Karmanos Cancer Institute cohort (Heath et al 2024) (n=57)
Patients with available pre-treatment PBMCs n=30:
Function assay
Available post-sip-T survival information (n=28)
Figure S1. Consort diagram. (A) Patients with available PBMCs, survival information, and self-reported race from the PRIME subset study of the PROCEED registry were eligible for this study. All PBMCs were tested in an in vitro functional assay (n=106), with qPCR SNP array performed on n=103 with sufficient recovered DNA. Comparisons of in vitro functional assay outputs with other pre-existing ELISpot, ELISA, and vaccine manufacture quality control (CD54 upreg and TNC) data sets were performed according to the availability of these metrics matched to each patient from the parent PRIME study. (B) Patients with available pre-sip-T treatment PBMCs, survival information, and self-reported race were used to validate findings in the PBMC function assays.
