# Supplementary material for: Peripheral Blood IFN Responses to Toll-Like Receptor 1/2 Signaling Associate with Longer Survival in Men with Metastatic Prostate Cancer Treated with Sipuleucel-T
Source: Cancer Res Commun. 2024 Oct 18;4(10):2724–33. doi: 10.1158/2767-9764.CRC-24-0439 (PMC11487532; doi:10.1158/2767-9764.CRC-24-0439)
Supplement: Figure S2 — Related to Figure 2 [file crc-24-0439_figure_s2_suppsf2.pptx]

## Slide 1
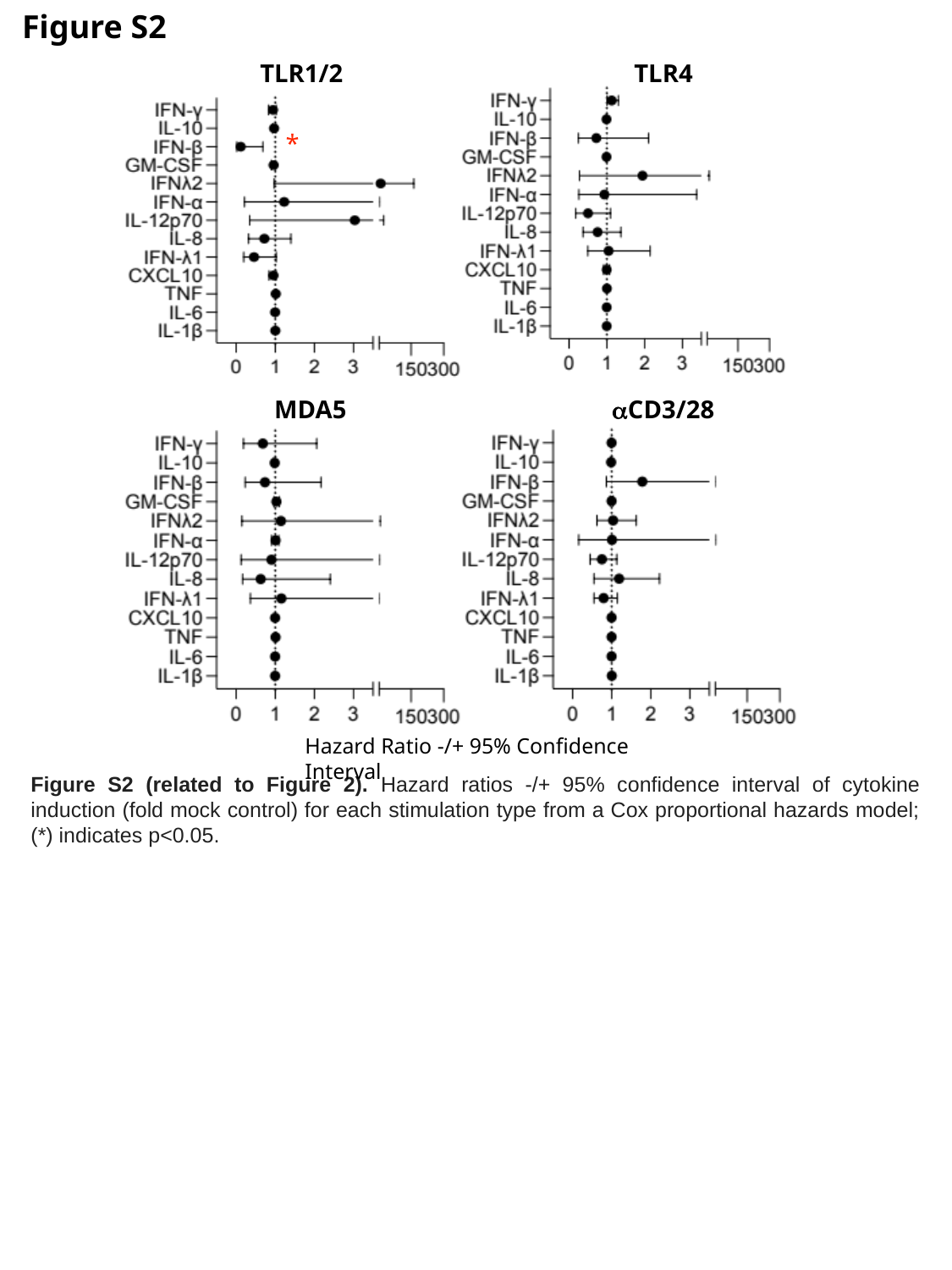

Figure S2
TLR4
TLR1/2
*
MDA5
aCD3/28
Hazard Ratio -/+ 95% Confidence Interval
Figure S2 (related to Figure 2). Hazard ratios -/+ 95% confidence interval of cytokine induction (fold mock control) for each stimulation type from a Cox proportional hazards model; (*) indicates p<0.05.
