# Supplementary material for: Peripheral Blood IFN Responses to Toll-Like Receptor 1/2 Signaling Associate with Longer Survival in Men with Metastatic Prostate Cancer Treated with Sipuleucel-T
Source: Cancer Res Commun. 2024 Oct 18;4(10):2724–33. doi: 10.1158/2767-9764.CRC-24-0439 (PMC11487532; doi:10.1158/2767-9764.CRC-24-0439)
Supplement: Figure S3 — Related to Figure 2 [file crc-24-0439_figure_s3_suppsf3.pptx]

## Slide 1
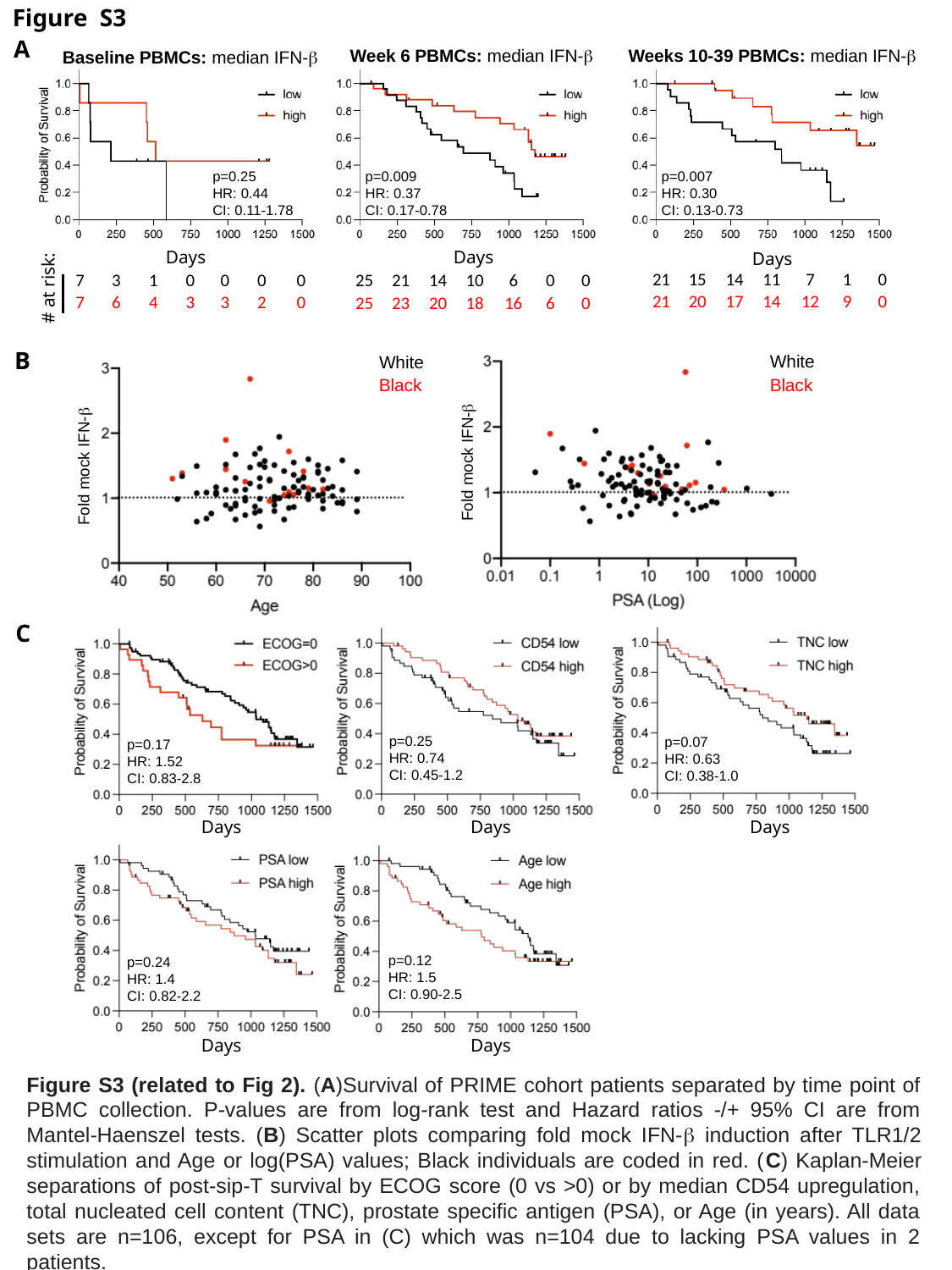

Figure S3
A
Week 6 PBMCs: median IFN-b
Weeks 10-39 PBMCs: median IFN-b
Baseline PBMCs: median IFN-b
p=0.007
HR: 0.30
CI: 0.13-0.73
p=0.25
HR: 0.44
CI: 0.11-1.78
p=0.009
HR: 0.37
CI: 0.17-0.78
Days
Days
Days
| 21 | 15 | 14 | 11 | 7 | 1 | 0 |
| --- | --- | --- | --- | --- | --- | --- |
| 21 | 20 | 17 | 14 | 12 | 9 | 0 |
| 7 | 3 | 1 | 0 | 0 | 0 | 0 |
| --- | --- | --- | --- | --- | --- | --- |
| 7 | 6 | 4 | 3 | 3 | 2 | 0 |
| 25 | 21 | 14 | 10 | 6 | 0 | 0 |
| --- | --- | --- | --- | --- | --- | --- |
| 25 | 23 | 20 | 18 | 16 | 6 | 0 |
# at risk:
B
White
White
Black
Black
Fold mock IFN-b
Fold mock IFN-b
C
p=0.25
HR: 0.74
CI: 0.45-1.2
p=0.07
HR: 0.63
CI: 0.38-1.0
p=0.17
HR: 1.52
CI: 0.83-2.8
Days
Days
Days
p=0.12
HR: 1.5
CI: 0.90-2.5
p=0.24
HR: 1.4
CI: 0.82-2.2
Days
Days
Figure S3 (related to Fig 2). (A)Survival of PRIME cohort patients separated by time point of PBMC collection. P-values are from log-rank test and Hazard ratios -/+ 95% CI are from Mantel-Haenszel tests. (B) Scatter plots comparing fold mock IFN-b induction after TLR1/2 stimulation and Age or log(PSA) values; Black individuals are coded in red. (C) Kaplan-Meier separations of post-sip-T survival by ECOG score (0 vs >0) or by median CD54 upregulation, total nucleated cell content (TNC), prostate specific antigen (PSA), or Age (in years). All data sets are n=106, except for PSA in (C) which was n=104 due to lacking PSA values in 2 patients.
