# Supplementary material for: Peripheral Blood IFN Responses to Toll-Like Receptor 1/2 Signaling Associate with Longer Survival in Men with Metastatic Prostate Cancer Treated with Sipuleucel-T
Source: Cancer Res Commun. 2024 Oct 18;4(10):2724–33. doi: 10.1158/2767-9764.CRC-24-0439 (PMC11487532; doi:10.1158/2767-9764.CRC-24-0439)
Supplement: Figure S4 — Related to Figure 3 [file crc-24-0439_figure_s4_suppsf4.pptx]

## Slide 1
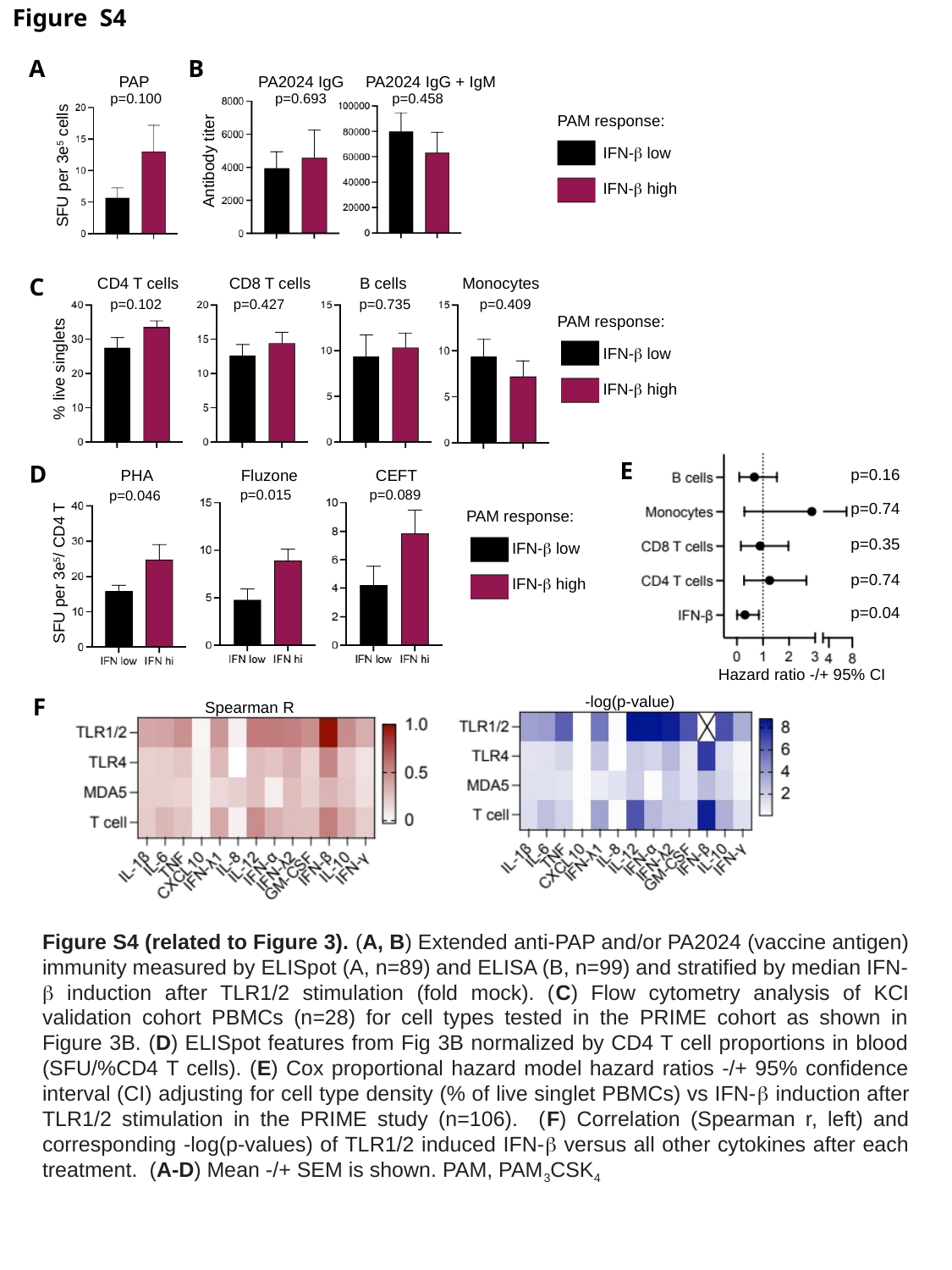

Figure S4
A
B
PAP
PA2024 IgG
PA2024 IgG + IgM
p=0.100
p=0.693
p=0.458
PAM response:
IFN-b low
Antibody titer
SFU per 3e5 cells
IFN-b high
C
CD4 T cells
CD8 T cells
B cells
Monocytes
p=0.102
p=0.427
p=0.735
p=0.409
PAM response:
IFN-b low
% live singlets
IFN-b high
E
D
p=0.16
PHA
Fluzone
CEFT
p=0.015
p=0.089
p=0.046
p=0.74
PAM response:
p=0.35
IFN-b low
SFU per 3e5/ CD4 T
p=0.74
IFN-b high
p=0.04
Hazard ratio -/+ 95% CI
-log(p-value)
F
Spearman R
Figure S4 (related to Figure 3). (A, B) Extended anti-PAP and/or PA2024 (vaccine antigen) immunity measured by ELISpot (A, n=89) and ELISA (B, n=99) and stratified by median IFN-b induction after TLR1/2 stimulation (fold mock). (C) Flow cytometry analysis of KCI validation cohort PBMCs (n=28) for cell types tested in the PRIME cohort as shown in Figure 3B. (D) ELISpot features from Fig 3B normalized by CD4 T cell proportions in blood (SFU/%CD4 T cells). (E) Cox proportional hazard model hazard ratios -/+ 95% confidence interval (CI) adjusting for cell type density (% of live singlet PBMCs) vs IFN-b induction after TLR1/2 stimulation in the PRIME study (n=106). (F) Correlation (Spearman r, left) and corresponding -log(p-values) of TLR1/2 induced IFN-b versus all other cytokines after each treatment. (A-D) Mean -/+ SEM is shown. PAM, PAM3CSK4
