# Supplementary material for: Peripheral Blood IFN Responses to Toll-Like Receptor 1/2 Signaling Associate with Longer Survival in Men with Metastatic Prostate Cancer Treated with Sipuleucel-T
Source: Cancer Res Commun. 2024 Oct 18;4(10):2724–33. doi: 10.1158/2767-9764.CRC-24-0439 (PMC11487532; doi:10.1158/2767-9764.CRC-24-0439)
Supplement: Figure S5 — Related to Figure 3 [file crc-24-0439_figure_s5_suppsf5.pptx]

## Slide 1
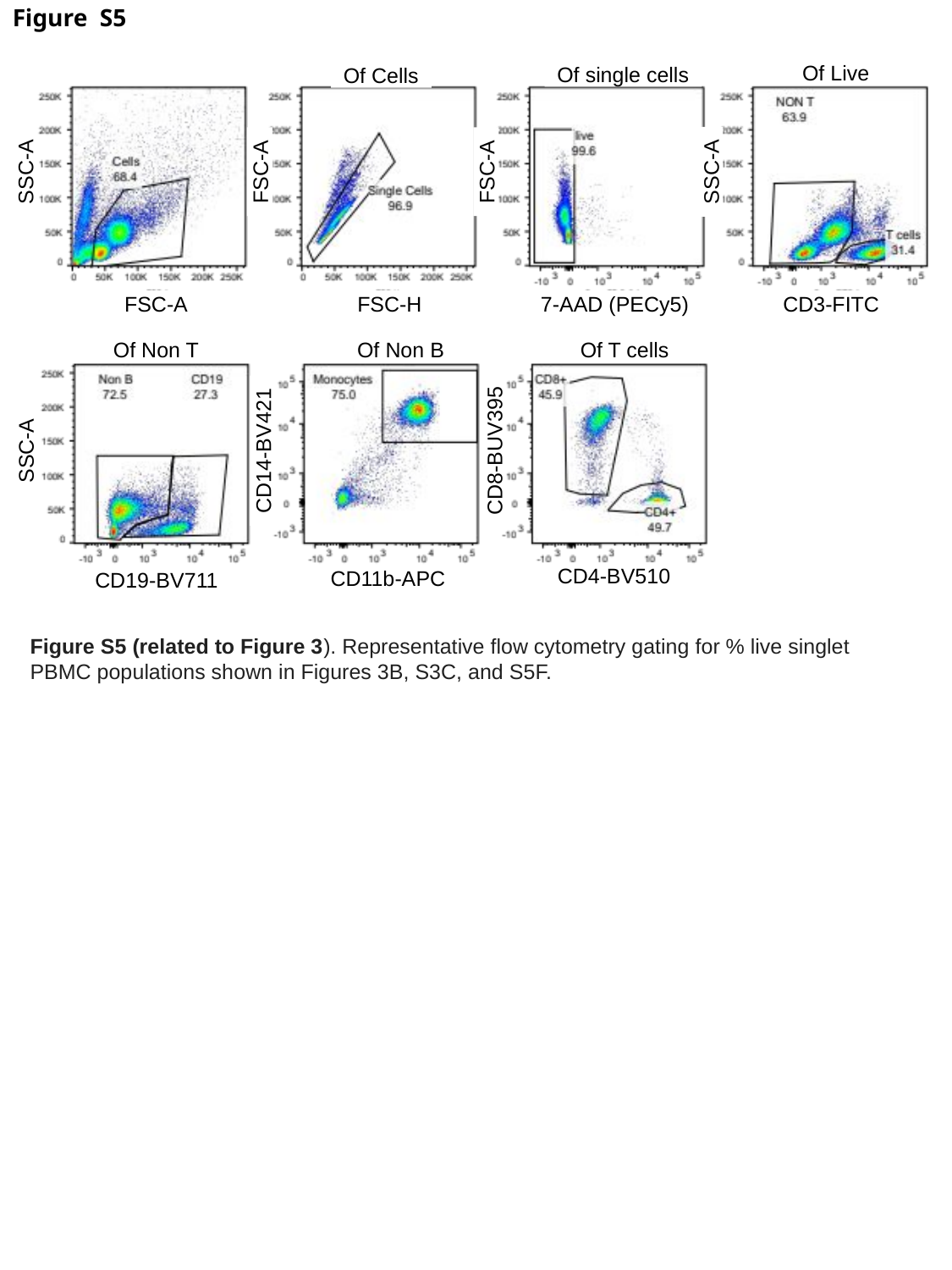

Figure S5
Of Live
Of single cells
Of Cells
SSC-A
FSC-A
FSC-A
SSC-A
FSC-A
FSC-H
7-AAD (PECy5)
CD3-FITC
Of Non T
Of Non B
Of T cells
CD8-BUV395
SSC-A
CD14-BV421
CD4-BV510
CD11b-APC
CD19-BV711
Figure S5 (related to Figure 3). Representative flow cytometry gating for % live singlet PBMC populations shown in Figures 3B, S3C, and S5F.
