# Supplementary material for: Peripheral Blood IFN Responses to Toll-Like Receptor 1/2 Signaling Associate with Longer Survival in Men with Metastatic Prostate Cancer Treated with Sipuleucel-T
Source: Cancer Res Commun. 2024 Oct 18;4(10):2724–33. doi: 10.1158/2767-9764.CRC-24-0439 (PMC11487532; doi:10.1158/2767-9764.CRC-24-0439)
Supplement: Figure S6 — Related to Figure 4 [file crc-24-0439_figure_s6_suppsf6.pptx]

## Slide 1
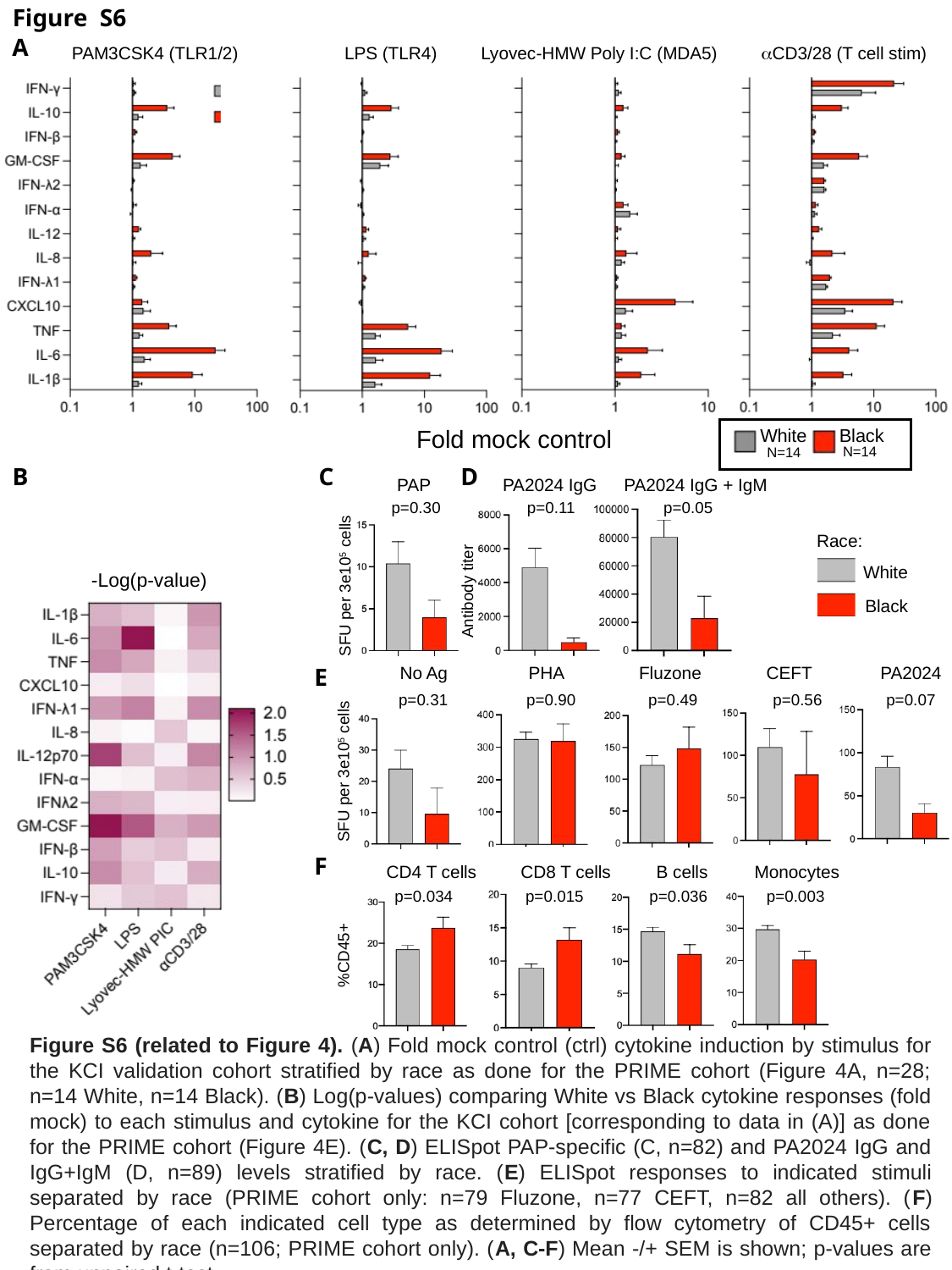

Figure S6
A
PAM3CSK4 (TLR1/2)
LPS (TLR4)
Lyovec-HMW Poly I:C (MDA5)
aCD3/28 (T cell stim)
Fold mock control
Black
White
N=14
N=14
B
C
D
PAP
PA2024 IgG
PA2024 IgG + IgM
p=0.05
p=0.30
p=0.11
Race:
White
-Log(p-value)
SFU per 3e105 cells
Antibody titer
Black
No Ag
PHA
Fluzone
CEFT
PA2024
E
p=0.31
p=0.90
p=0.49
p=0.56
p=0.07
SFU per 3e105 cells
F
CD4 T cells
CD8 T cells
B cells
Monocytes
p=0.034
p=0.015
p=0.036
p=0.003
%CD45+
Figure S6 (related to Figure 4). (A) Fold mock control (ctrl) cytokine induction by stimulus for the KCI validation cohort stratified by race as done for the PRIME cohort (Figure 4A, n=28; n=14 White, n=14 Black). (B) Log(p-values) comparing White vs Black cytokine responses (fold mock) to each stimulus and cytokine for the KCI cohort [corresponding to data in (A)] as done for the PRIME cohort (Figure 4E). (C, D) ELISpot PAP-specific (C, n=82) and PA2024 IgG and IgG+IgM (D, n=89) levels stratified by race. (E) ELISpot responses to indicated stimuli separated by race (PRIME cohort only: n=79 Fluzone, n=77 CEFT, n=82 all others). (F) Percentage of each indicated cell type as determined by flow cytometry of CD45+ cells separated by race (n=106; PRIME cohort only). (A, C-F) Mean -/+ SEM is shown; p-values are from unpaired t-test.
