# Supplementary material for: Peripheral Blood IFN Responses to Toll-Like Receptor 1/2 Signaling Associate with Longer Survival in Men with Metastatic Prostate Cancer Treated with Sipuleucel-T
Source: Cancer Res Commun. 2024 Oct 18;4(10):2724–33. doi: 10.1158/2767-9764.CRC-24-0439 (PMC11487532; doi:10.1158/2767-9764.CRC-24-0439)
Supplement: Figure S7 — Related to Figure 5 [file crc-24-0439_figure_s7_suppsf7.pptx]

## Slide 1
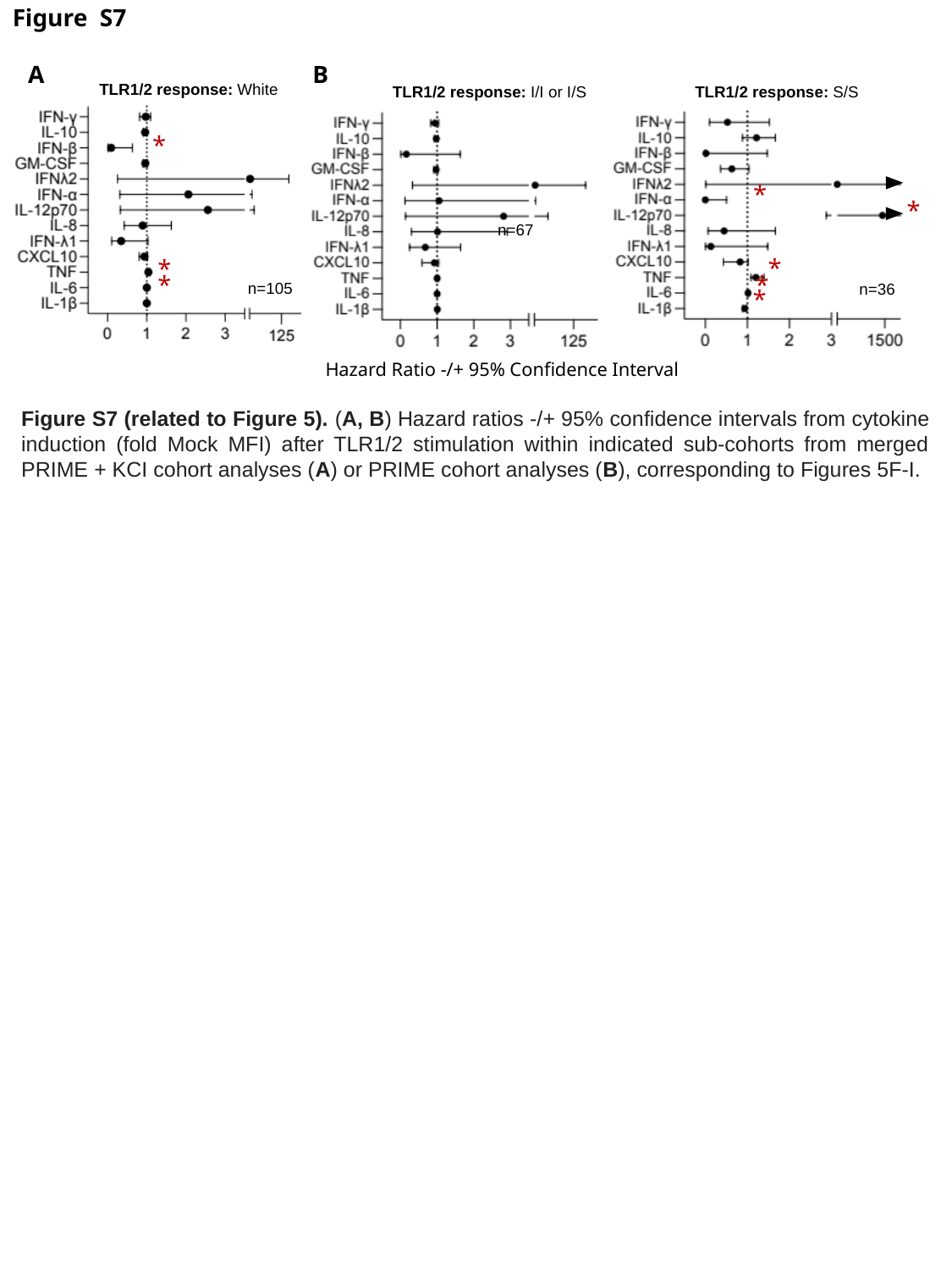

Figure S7
A
B
TLR1/2 response: White
TLR1/2 response: I/I or I/S
TLR1/2 response: S/S
*
*
*
n=105
*
*
*
*
n=36
*
n=67
Hazard Ratio -/+ 95% Confidence Interval
Figure S7 (related to Figure 5). (A, B) Hazard ratios -/+ 95% confidence intervals from cytokine induction (fold Mock MFI) after TLR1/2 stimulation within indicated sub-cohorts from merged PRIME + KCI cohort analyses (A) or PRIME cohort analyses (B), corresponding to Figures 5F-I.
